# Editorial Note: Tracking the Luminal Exposure and Lymphatic Drainage Pathways of Intravaginal and Intrarectal Inocula Used in Nonhuman Primate Models of HIV Transmission

**DOI:** 10.1371/journal.pone.0298985

**Published:** 2024-02-12

**Authors:** The PLOS ONE Editors

Following publication of this article [1] and its subsequent Correction [2], questions were raised about the SIV status of the macaques at the time of the study procedures.

As stated in the Materials and Methods section “Prior to use, all animals were tested and confirmed seronegative for macacine herpes virus 1 (herpes B), Simian Immunodeficiency Virus (SIV), Simian T Lymphotropic Virus (STLV), and Simian Retrovirus (SRV) and were negative for SRV by PCR as well.”

The corresponding author has clarified that testing and seronegative status was confirmed at the time of entry to the animal facility, before use in research, but that the study utilized animals that were used in other studies, during which some of the animals were experimentally infected or challenged with SIV/SHIV. No animals were SIV/SHIV infected during the MRI phase of the study, but 19 of the animals were SIV/SHIV infected at the time of the necropsy and methylene blue dye distribution experiment.

Individual-level infection status information for the 24 study animals is provided here as S2 File.

The corresponding author has stated that the SIV status does not affect dye uptake results or the conclusions of the study; a member of the *PLOS ONE* Editorial Board reviewed the additional information regarding infection status and advised that this does not affect support for the study’s results and conclusions.

The earlier Correction [2] corrected an error in one of the reported animal IDs in Fig 1 (animal ID 4450 is incorrect; the correct ID is 4550). Readers are informed that this ID number correction also applies to the second paragraph of the Results section of the article [1].

## Supporting information

S2 File SIV status information. (PDF)
