# Supplementary material for: Editorial Note: Tracking the Luminal Exposure and Lymphatic Drainage Pathways of Intravaginal and Intrarectal Inocula Used in Nonhuman Primate Models of HIV Transmission
Source: PLoS One. 2024 Feb 12;19(2):e0298985. doi: 10.1371/journal.pone.0298985 (PMC10861071; doi:10.1371/journal.pone.0298985)
Supplement: S2 File — (PDF) [file pone.0298985.s001.pdf]

Animal I.D.	Gender	Infection with SIV or SHIV	Days post infection to necropsy	Rectum Intraluminal *MB 1ml (n=5)	Rectum Intraluminal *MB 3ml (n=6)	Vagina Intraluminal *MB (n=6)	Rectum Submucosal *MB (n=5)	Colon Submucosal *MB (n=5)	Vagina Submucosal *MB (n=2)	Rectum Intraluminal MRI 1ml (n=3)	Rectum Intraluminal MRI 3ml (n=2)	Vagina Intraluminal MRI (n=3)	Rectum Submucosal MRI (n=4)	Colon Submucosal MRI (n=3)	Vagina Submucosal MRI (n=2)	Vaginal Infection (n=1)
4550^	M	exposed uninfected	N/A	√												
4543	M	SIV	14 days	√												
4317	M	SIV	13 days		√											
4324	M	SIV	13 days		√											
HV9	F	SIV	13 days		√											
CF86	M	exposed uninfected	N/A		√											
986	F	SIV	16 days			√										
ZB35	F	SIV	5 days		√	√										
BB78	F	SIV	13 days	√		√										
ZE72	F	SIV	5 days	√		√										
DC66	F	Infected with SIV after MRI	7 days	√		√						√			√	
ZG04	F	Infected with SIV after MRI	7 days		√	√				√	√	√	√	√	√	
ZG83	M	Uninfected	N/A							√			√			
ZJ04	M	Uninfected	N/A								√		√	√		
ZJ47^^	M	Uninfected	N/A										√	√		
ZJ63	F	Infected with SIV after MRI	140 days						√	√		√				
ZD28	F	SIV	140 days						√							
ZH08	M	SHIV	208 days				√	√								
ZH39	M	SHIV	215 days					√								
Zi23	M	SHIV	217 days				√	√								
ZG20	M	SHIV	217 days				√	√								
Zi47	M	SHIV	196 days				√									
Zi52	M	SHIV	196 days				√	√								
A7E018	F	SIV	3 days													√

* Methylene blue dye administration

^ "4550" is correct animal ID. Incorrectly identified as "4450" in Figure 1.

^^ "ZJ47" is correct animal ID. Incorrectly identified as "ZJ49" and "49" in Table 1.

Two series of Fenbendazole + Paromomycin (50mg/kg PO BID x 12 days)
